# S-PRG-based toothpastes compared to NaF toothpaste and NaF varnish on dentin permeability *in vitro*

**DOI:** 10.1590/1678-7757-2022-0082

**Published:** 2022-06-06

**Authors:** Victor MOSQUIM, Giovanna Speranza ZABEU, Gerson Aparecido FORATORI-JUNIOR, Alessandra Buhler BORGES, Daniela RIOS, Ana Carolina MAGALHÃES, Linda WANG

**Affiliations:** 1 Universidade de São Paulo Faculdade de Odontologia de Bauru Departamento de Dentística, Endodontia e Materiais Odontológicos Bauru SP Brasil Universidade de São Paulo , Faculdade de Odontologia de Bauru , Departamento de Dentística, Endodontia e Materiais Odontológicos , Bauru , SP , Brasil .; 2 Centro Universitário UniSagrado Faculdade de Odontologia Bauru SP Brasil Centro Universitário UniSagrado , Faculdade de Odontologia , Bauru , SP , Brasil .; 3 Universidade de São Paulo Faculdade de Odontologia de Bauru Departamento de Odontopediatria, Ortodontia e Saúde Coletiva Bauru SP Brasil Universidade de São Paulo , Faculdade de Odontologia de Bauru , Departamento de Odontopediatria, Ortodontia e Saúde Coletiva , Bauru , SP , Brasil .; 4 Universidade Estadual Paulista Instituto de Ciência e Tecnologia Departamento de Odontologia Restauradora São José dos Campos SP Brasil Universidade Estadual Paulista - UNESP, Instituto de Ciência e Tecnologia , Departamento de Odontologia Restauradora , São José dos Campos , SP , Brasil .; 5 Universidade de São Paulo Faculdade de Odontologia de Bauru Departamento de Ciências Biológicas Bauru SP Brasil Universidade de São Paulo , Faculdade de Odontologia de Bauru , Departamento de Ciências Biológicas , Bauru , SP , Brasil .

**Keywords:** Dentin, Dentin permeability, Dentin sensitivity, Fluorides, Toothpastes

## Abstract

**Objectives:**

To analyze the effect of 5 toothpastes containing different percentages of S-PRG fillers compared to NaF toothpaste and NaF varnish on the dentin hydraulic conductance (Lp).

**Methodology:**

Dentin disks (1.0±0.2 mm thickness) were cut from third molars, and their Lp values were evaluated using Flodec. The specimens were allocated into 7 groups (n=8). The minimum (smear layer) and the maximum (after acid etching) Lp values were recorded. Lp was also assessed after treatment with either a 0wt.%, 1wt.%, 5wt.%, 20wt.%, or 30wt.% S-PRG toothpaste, a NaF toothpaste, or a NaF varnish. Toothpastes were applied by brushing for 15 s, allowing it to settle for 1 min, and rinsing with deionized water. The NaF varnish was applied for 4 min and was removed with a probe. Specimens were exposed to citric acid (6%, pH 2.1, 1 min) and their final Lp was recorded. The pH of all products was recorded (n=3) and specimens from each group were analyzed by Laser Scanning Confocal Microscopy (LSCM). Data were subjected to 2-way repeated measures ANOVA and post-hoc Bonferroni (a=0.05).

**Results:**

The highest Lp reduction was noticed for the 5wt.% S-PRG toothpaste, NaF toothpaste, and NaF varnish. However, the toothpastes containing 5wt.%, 20wt.%, and 30wt.% of S-PRG were similar to all toothpastes but differed from the NaF varnish. After erosion, all groups retrieved their maximum Lp values, except for the NaF varnish. The LSCM evidenced deposits on the surface of specimens treated with 5%, 20%, and 30% S-PRG-based toothpastes and NaF toothpaste. Even more deposits were observed for the NaF varnish. After the erosive challenge, the deposits were diminished in all groups.

**Conclusion:**

Toothpastes containing 5wt.%, 20wt.%, and 30wt.% of S-PRG fillers behaved similarly to a conventional NaF toothpaste, even after an erosive challenge. The NaF varnish promoted better reduction of the Lp, but its effect was also diminished after erosion.

## Introduction

The Surface Pre-Reacted Glass (S-PRG) fillers are manufactured using milled pre-reactive fluoro-boro-aluminosilicate glass, which is treated with an alcoholic polysiloxane solution to create a porous external layer. This glass is then sprayed with a polyacrylic acid aqueous solution, creating an intermediate phase between the external treated surface and the inner glass core. ^1^

Due to its manufacturing technology, these trilaminar particles are able to interact with the external environment by releasing six different ions (Sr ^2^ , F ^-^ , SiO _3_^2-^ , Al ^3^ , BO _3_^3-^ and Na ^+^ ). ^2^ Thus, they offer multiple benefits when activated, such as anti-plaque effect and biofilm control, ^3 , 4^ reduction of the caries risk, ^2 , 5^ fluoride release and recharge, ^6^ in addition to its ability to prevent demineralization and improve remineralization. ^7 - 12^ However, the studies that tested the efficacy of the S-PRG fillers are mainly restricted to enamel, ^7 , 9 - 12^ in which the chemical and morphological composition differ from the underlying dentin.

The dentin is a mineralized tissue morphologically constituted by tubules, which vary in number and diameter according to the proximity of the pulp. ^13^ These tubules are usually filled with fluids and extensions of odontoblastic and nerve cells found in the dental pulp. When dentin is exposed to the oral environment, chemical, thermal, tactile, and osmotic stimuli excite these nerves and generate a painful response known as dentin hypersensitivity. ^14 - 18^

So far, there is no consensus on the real mechanism for dentinal sensitivity. The most accepted theory is the hydrodynamic theory formulated by Brännström and Åström ^14^ (1964). This theory assumes that these chemical, thermal, tactile, and osmotic stimuli can cause an abrupt movement of the fluid within the dentinal tubules and stimulate the intra-pulpal nerve cells, leading to dentin hypersensitivity. Therefore, most desensitizing materials work by suppressing the effect, reducing the diameter of the dentinal tubules and, consequently, the movement of dentinal fluids. ^17 , 19 - 23^ Among these materials, fluoridated salts are often used since fluoride allows the formation of calcium fluoride (CaF _2_ ) deposits. ^16 , 17 , 22 , 24 , 25^ Most of these products, however, are limited to the effects of time, especially due to erosive challenges within the oral cavity. Since the prevalence of dental erosion is increasing, approaches that can detain the consequences of tooth wear are desired. ^17 , 22 , 25^ Therefore, strategies that could provide long-lasting prevention and/or therapeutic efficacy to help control the impact of dentin hypersensitivity on the quality of life of individuals are needed.

Up to now, studies using the S-PRG fillers have focused mainly on evaluating their remineralizing effect on enamel samples in an artificial caries environment. The results mainly address the role of ions such as Sr ^2^ , which can reinforce the mineral structure of the enamel by forming the strontium-apatite; ^6 , 9^ F-, proving to be effective at reducing demineralization and promoting remineralization; ^7 - 11^ and Al ^3^ , which can promote tubule occlusion and reduce dentin permeability *in vitro* . ^26^

Considering these properties, it seems reasonable to suggest that this technology could potentially contribute to the relief of dentin hypersensitivity, given the multiple ions composing this material. Since dentin permeability is a validated way to assess this potential, it can serve as a method to infer this clinical ability. ^24^ Thus, this *in vitro* study aims to investigate the potential of experimental toothpastes containing S-PRG fillers in five different concentrations on dentin permeability and to compare their efficacy with a conventional NaF-based toothpaste and a NaF varnish. The tested null hypothesis is that there is no difference in dentin permeability after treatment with S-PRG-based toothpastes, NaF toothpaste, or a NaF varnish.

## Methodology

### Experimental design

This *in vitro* study analyzed the effect of five experimental toothpastes containing 0 wt.%, 1 wt.%, 5 wt.%, 20 wt.%, and 30 wt.% of S-PRG fillers on the reduction of human dentin hydraulic conductance (Lp) (dependent variable) compared to a 1,450 ppm NaF-based toothpaste and a 22,600 ppm NaF varnish (Duraphat, Colgate, New York, USA). The independent variables were: 1) material, in seven levels; and 2) dentin condition, in four levels (with smear layer, without smear layer, treated with one of those products, and after an erosive challenge).

### Toothpaste fabrication and pH analysis

The experimental toothpastes were fabricated by Shofu (Kyoto, Japan) and they were composed of silica, hydrated silica, carboxymethylcellulose sodium, glycerol, sorbitol solution, lime mint flavor, dipotassium glycyrrhizate, water, and S-PRG fillers, which differed among them in concentration, being 0 wt.% (negative control – no S-PRG fillers), 1 wt.%, 5 wt.%, 20 wt.% and 30 wt.%. The toothpaste containing 1,450 ppm of NaF was also fabricated using the abovementioned composition but with no S-PRG fillers (positive control).

A pH electrode (2A09E, Analyser, São Paulo, Brazil) calibrated with standard pH levels of 7.0 and 4.0 was used to measure the pH levels of the toothpastes as slurries (1:3 with deionized water) (n=3). ^27^ The pH of the NaF varnish was measured using a universal pH indicator strip (Merck, Darmstadt, Germany).

### Sample size estimation

Sample size was estimated using the G*Power 3.1 software (Aichach, Germany). Based on the results of the pilot study, the effect size was estimated to be 0.752, with an a=0.05 and power (1-b)=0.8. The correlation among repeated measures was set at 0.5. Based on this information, the total sample size estimated was 28 (n=4 per group).

However, based on a previously published study, ^24^ a sample size of n=8 per group was determined to achieve power (1-b)>0.9.

### Sample preparation

This study was approved by the local Ethics Committee. In total, 56 sound third molars were used, which were stored in 0.1% thymol solution (pH 7.0) during the experiment.

Crowns were sectioned using a diamond wafer blade (XL-12205, Extec, Enfield, USA) in an automatic Isomet machine (Buehler, Lake Bluff, USA) at 300 rpm and under cooling with deionized water. The teeth were cut transversely right below the amelodentin junction and above the pulp horns, resulting in 1.0±0.2 mm-thick discs. One single disk was obtained from each tooth. Thickness was controlled using a digital caliper (Digimatic Caliper Absolute, Mitutoyo, Kawasaki, Japan). When prepared, the specimens were stored in deionized water at 10 °C during the experiment.

### Treatment protocols

After cutting, the specimens were immersed in 37% phosphoric acid (pH 1.0) for 15 s, and rinsed with deionized water to remove the smear layer formed during cutting, allowing the measurement of the specimen’s maximum permeability values (LpMax). ^24^ Dentin permeability was evaluated as hydraulic conductance (Lp) using a measurement device (Flodec, DeMarco Engineering, Geneva, Switzerland). ^28^ The LpMax values were used to randomize and allocate the specimens into seven groups (n=8) allowing the baseline permeability to be similar among all groups.

Treatment was conducted using one of the seven materials after the specimens were gently dried with an absorbent paper. The toothpaste was applied with a disposable microbrush, by actively brushing for 15s, allowing it to settle for 1 min, and then rinsing with deionized water. The varnish was applied on the specimens by allowing it to settle for 4 min and then gently removed with an explorer probe.

### Dentin permeability measurement

The Flodec device contains a split chamber with two o-rings to allow water passage only to a standardized circular area of each specimen (16.9 mm ^2^ ). The split chamber is attached to a capillary tube containing an air bubble (internal diameter: 0.83 mm; external 4 mm; detectable volume 2.71 nL) and a water column with a reservoir. The water reservoir was filled with deionized water at room temperature and placed 140 cm above the Flodec to standardize the pressure at 2 psi. Only one specimen was analyzed at a time. The specimens were treated with their assigned product without being removed from the chamber, so no differences in their positions could have influenced their Lp values.

Lp was measured for 5 min based on the movement of the air bubble inside the capillary glass tube. The first 2 min were disregarded, and the final 3 min were averaged to obtain the values of Lp for each experimental condition.

### Dentin condition analyses

Four consecutive readings of Lp for each sample were conducted during the study, being one for each dentin condition, ^24^ as described:

Minimum permeability (LpMin): with the smear layer, which was created by standardized polishing of the occlusal surface of each dentin disc with #600 silicon carbide (SiC) sandpapers (Buehler, Lake Bluff, USA); ^29^

Maximum permeability (LpMax): after removal of smear layer by applying 37% phosphoric acid gel for 15 s on the occlusal surface of each specimen followed by rinsing with deionized water;

Treatment permeability (LpTreat): the surface of each specimen was gently dried with an absorbent paper and treated with one of the tested materials, and then rinsed with deionized water.

Permeability after erosive challenge (LpEro): after treatment, the specimens were subjected to an erosive challenge (6% citric acid, pH 2.1, 1 min), then rinsed with deionized water.

As conducted in a previously published study, ^30^ the LpMax value was considered as 100% permeability; the LpMin, LpTreat, and LpEro were estimated based the LpMax value (e.g. if LpMax was 20 µLmin ^-1^ , it was considered to be 100%, so the value of 1 was given. If, after treatment with one of the designated products, the LpTreat value was 5 µLmin ^-1^ , the LpTreat would be estimated at 25% of LpMax, so the value of 0.25 would be attributed to LpTreat). This method allowed for the estimation of the specimen’s Lp in comparison to itself before the application of the products, allowing each specimen to be used as its own control group.

### Laser scanning confocal microscopy

Specimens of each group were also evaluated by a laser scanning confocal microscope (LSCM) (Leica TCS SPE, Leica Microsystems, Mannheim, Germany). For this analysis, 15 new dentin disks were prepared and a central groove was made using a spherical carbide bur (KG Sorensen, São Paulo, Brazil) to divide the surface into two halves. ^30^ The specimens were prepared exactly as performed in the “dentin conditions analyses” section, but one half represented the dentin after treatment with one of the designated product (pTreat), while the other was immersed in citric acid to represent the dentin after the 1-min erosive challenge (pEro) (n=2/group). One specimen represented the dentin with (pMin) and without smear layer (pMax) and was used as a control for all groups.

Reflectance light and fluorescent light were used to obtain images from the dentin surface evidencing the presence of solid deposits and the natural fluorescence of dentin, respectively. The specimens were analyzed both in the XY axis and in the XZ axis, in a standardized depth of 20 µm from the surface. The resulting images from reflectance and fluorescent illumination were superposed to allow the visualization of the deposits on the dentin surface and within the dentinal tubules. ^30^

### Statistical analysis

The average of each Lp reading was organized in Microsoft Excel (Microsoft Corp., Redmond, WA, USA) spreadsheets and the LpMin, LpTreat, and LpEro were transformed in percentage levels proportional to the LpMax value, which was considered as 100%. The data were transformed in Log _10_ to allow normal distribution and homogeneity. Two-way repeated measures ANOVA and *post-hoc* Bonferroni tests were used for comparison among the Materials and Dentin Conditions factors (a=0.05). These tests were performed using IBM SPSS Statistics v23.0.0 (IBM Corp, Armonk, New York, USA). The average of pH analysis was estimated for each material using Microsoft Excel.

## Results

The pH of all toothpastes ranged from 7.21 to 7.63, and the pH of the NaF varnish was 4.0. Table 1 shows the means and standard deviation of each tested toothpaste.

**Table 1:** Mean ± standard deviation of pH of each tested material

Product	pH
0 wt.% S-PRG	7.45±0.04
1 wt.% S-PRG	7.33±0.02
5 wt.% S-PRG	7.25±0.01
20 wt.% S-PRG	7.21±0.02
30 wt.% S-PRG	7.26±0.03
NaF toothpaste	7.63±0.07
NaF varnish	4.00

**Figure 1:**
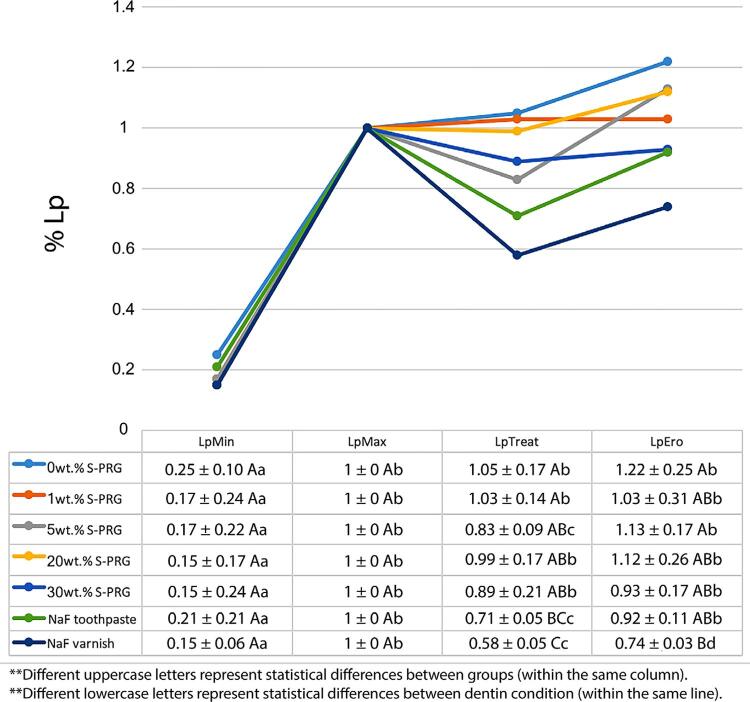
Mean ± standard deviation of the percentage of hydraulic conductance (% Lp) for each tested material

Statistical analysis evidenced that both Material (p=0.009) and Dentin (p<0.001) factors were statistically significant, as well as their interaction (Material*Dentin, p<0.05). No statistical differences were seen between groups for both LpMin and LpMax ( Figure 1 ), evidencing the standardized condition for all groups, avoiding any bias. For all groups, LpMin was statistically different from LpMax.

The highest values of LpTreat were seen for the toothpastes containing 0 wt.% and 1 wt.% of S-PRG fillers, which differed from the NaF toothpaste and varnish. The toothpastes containing 5 wt.%, 20 wt.%, and 30 wt.% of S-PRG were similar to all toothpastes but differed from the NaF varnish. The specimens treated with the NaF varnish showed the lowest LpTreat value.

Regarding LpEro, it significantly differed from LpMin, but it did not differ from LpMax, for all tested toothpastes. The highest LpEro values were seen for the groups with 0 wt.% and 5 wt.% of S-PRG fillers, which were similar to all groups, except for the group in which the NaF varnish was applied. The groups with 1 wt.%, 20 wt.%, and 30 wt.% were similar to the values of the NaF toothpaste group and the NaF varnish group. The lowest values of LpEro were seen for the varnish group.

Only the groups treated with the 5 wt.% S-PRG-based toothpaste, NaF toothpaste, and NaF varnish could present a significant reduction in the permeability levels when compared with their respective LpMax values. However, after the erosive challenge, only the group treated with the NaF varnish was able to keep a LpEro significantly different from the LpMax value.

**Figure 2:**
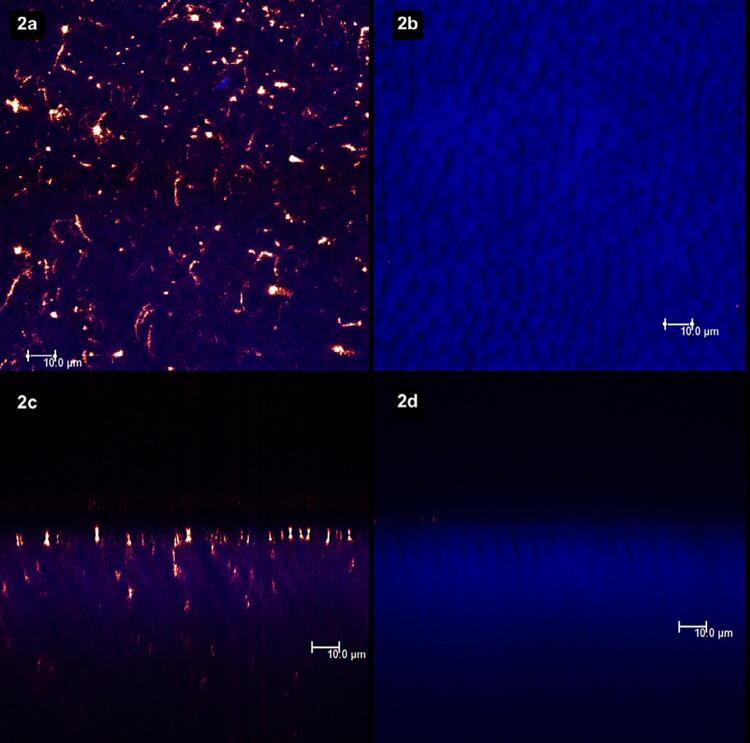
A smear layer can be seen on the dentin surface (2a) and at the aperture of the dentinal tubules (2c). After etching with 37% phosphoric acid, the smear layer was removed from the surface (2b) and from the dentinal tubules (2d)

**Figure 3:**
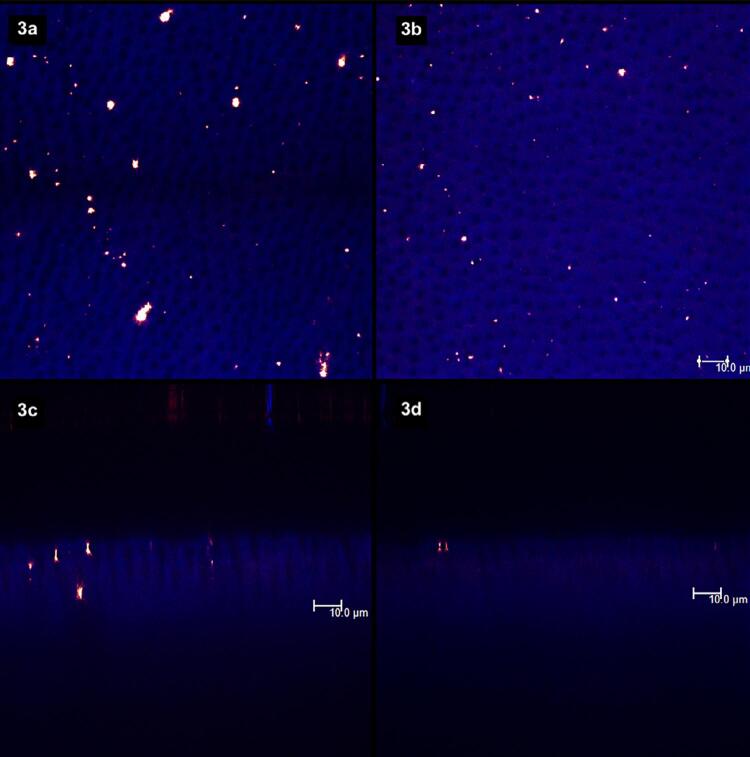
After the application of a toothpaste without S-PRG fillers, the dentin surface (3a) and the aperture of dentinal tubules (3c) presented few solid deposits, which were almost completely removed from the surface (3b) and from the tubules (3d) after the erosive challenge

**Figure 9:**
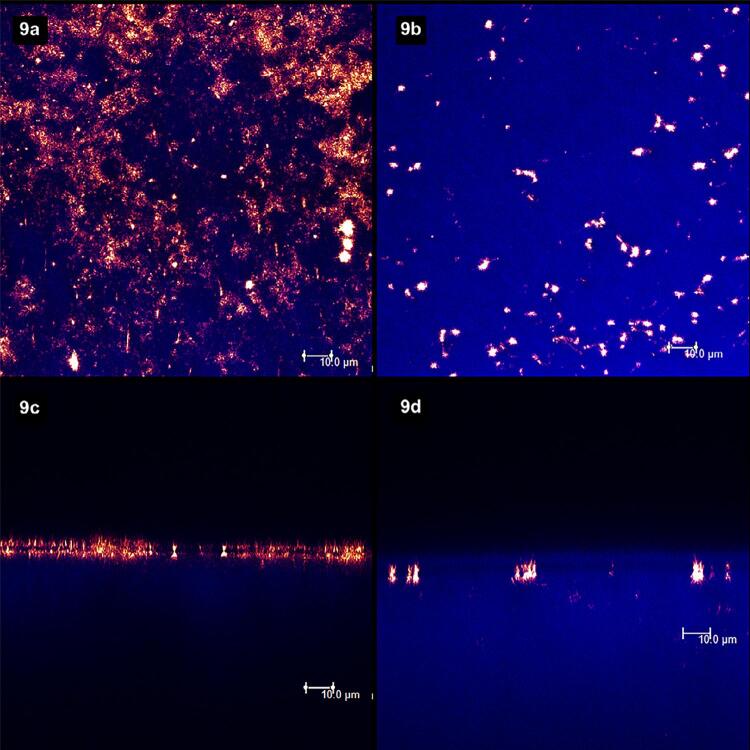
The application of the 22,600 ppm NaF varnish resulted in many solid deposits on the surface (9a) and at the aperture of the tubules (9c), but they were significantly diminished after the erosive challenge, both on the surface (9b) and at the tubules’ apertures (9d).

**Figure 4:**
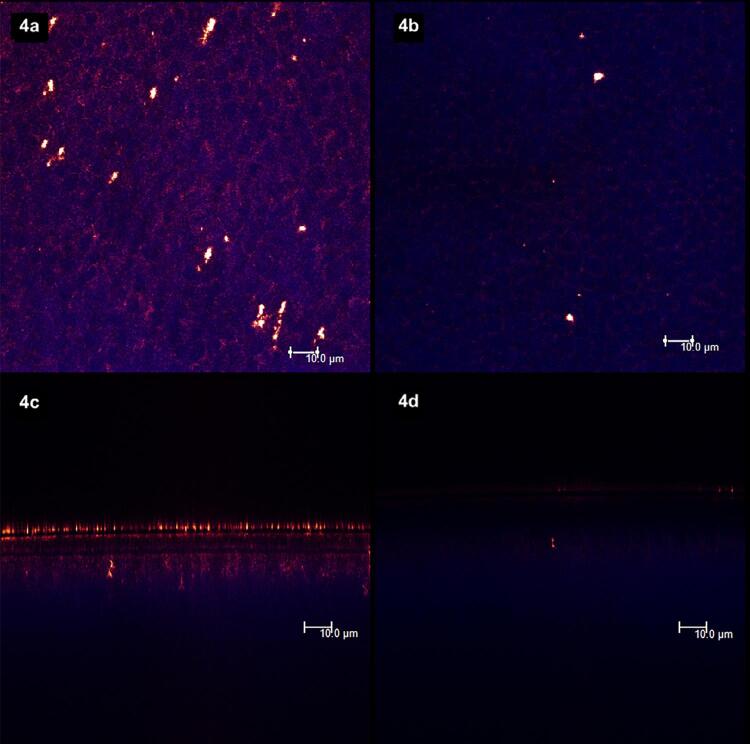
The toothpaste containing 1 wt.% of S-PRG fillers could not promote the formation of solid deposits on the surface (4a) and at the aperture (4c) of dentinal tubules. After the acid challenge, the surface (4b) and the aperture of the tubules (4d) were mostly free of deposits

**Figure 5:**
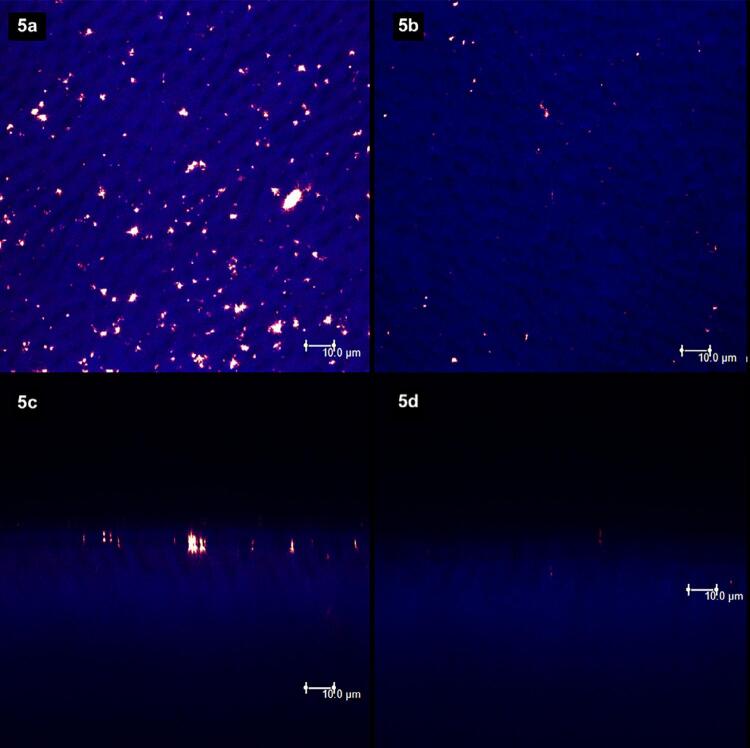
The toothpaste with 5 wt.% of S-PRG was able to promote the formation of some solid deposits on the dentin surface (5a) and at the aperture of dentinal tubules (5c). After the erosive challenge, however, these deposits were almost completely removed from the surface (5b) and from the dentinal tubules (5d)

**Figure 6:**
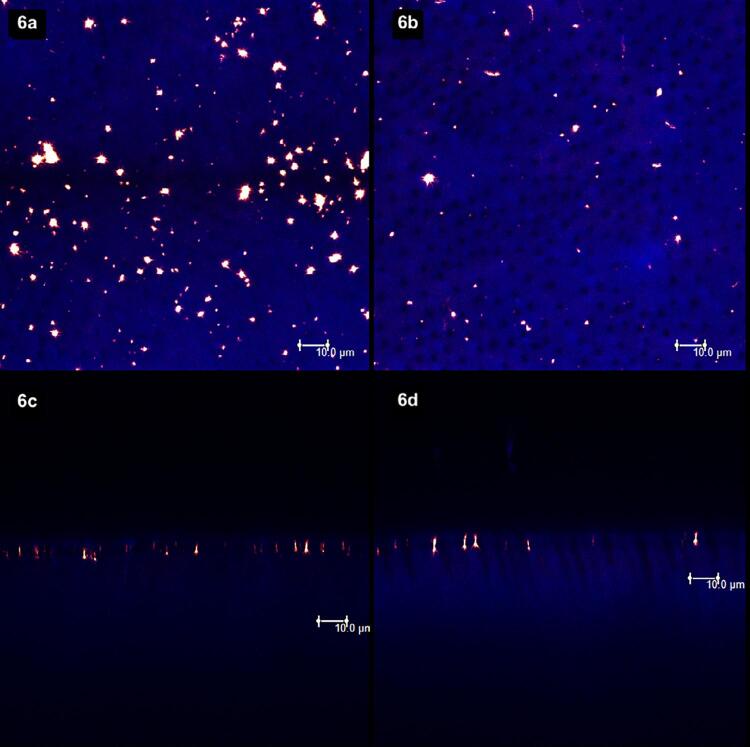
The 20 wt.% S-PRG-based toothpaste promoted some solid deposits on the dentin surface (6a) and at the dentinal tubules (6c). Yet, the number of particles on the surface (6b) and at the aperture of the tubules (6d) decreased significantly after the erosive challenge

**Figure 7:**
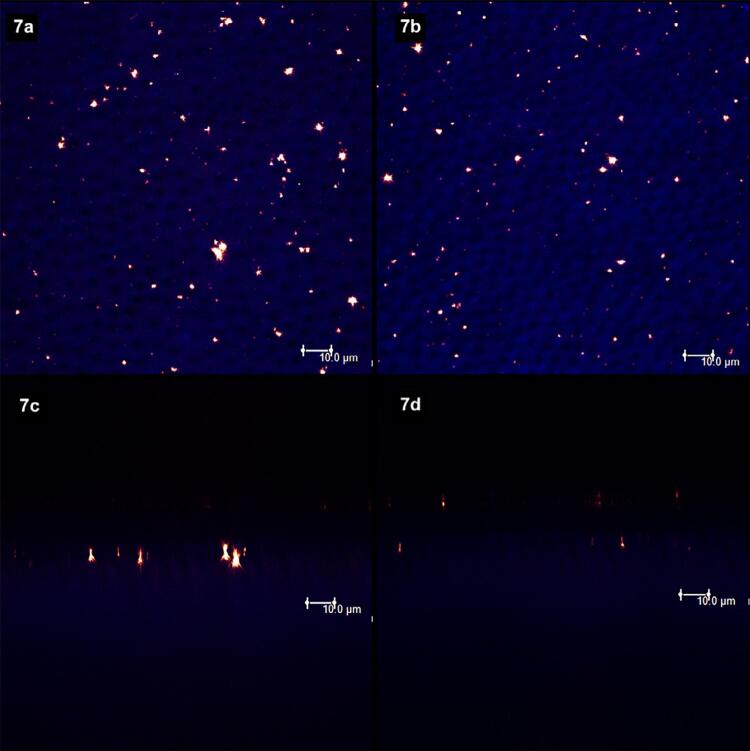
Few solid deposits are seen on the dentin surface (7a) and the aperture of tubules (7c) after the application of the 30 wt.% S-PRG-based toothpaste. After the erosive challenge, the number and size of particles on the surface (7b) and at the aperture (7d) decreased

**Figure 8:**
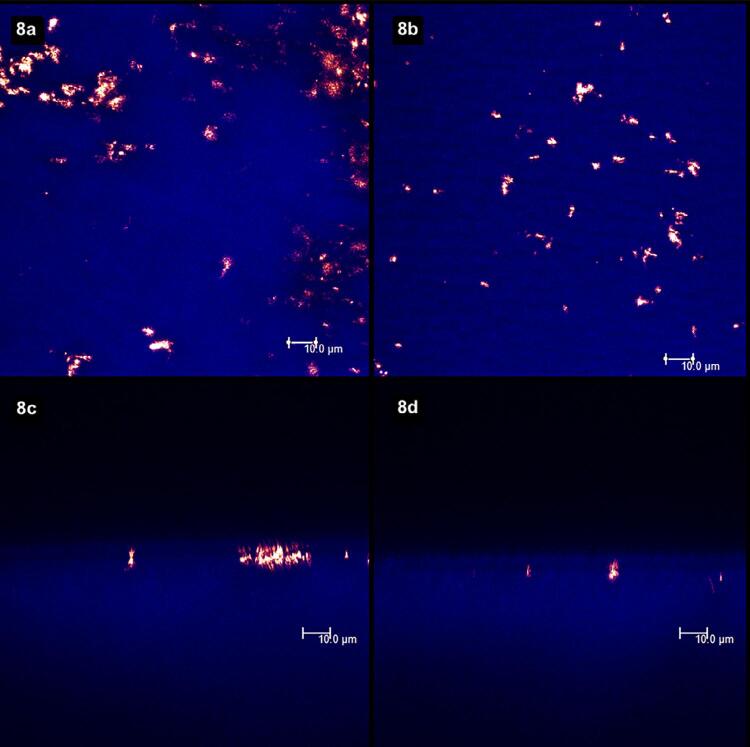
The application of a toothpaste containing 1,450 ppm NaF resulted in the formation of some deposits on the dentin surface (8a) and at the aperture of dentinal tubules (8c). After the erosive challenge, the number

Figures 2-9 show laser scanning confocal microscopic (LSCM) images. Figures “a” and “b” were obtained in the XY axis, while figures “c” and “d” were obtained in the XZ axis. Each figure corresponds to the superposition of images obtained under fluorescent and reflective lights. Figure 2 represents the specimens with (left column, figures “a” and “c”) and without (right column, figures “b” and “d”) smear layer. From Figure 3 to Figure 9 , the left column (figures “a” and “c”) represents the specimens after being treated with the designated product (LpTreat), and the figures in the right column (figures “b” and “d”), the specimens after the erosive challenge (LpEro).

The specimen in Figure 2a is covered by smear layer, and this layer also seems to occlude the aperture of the dentinal tubules ( Figure 2c ). After a 15-second acid etching, the smear layer was removed from the dentin surface ( Figure 2b ) and the aperture of the tubules is seen unclogged ( Figure 2d ).

When the specimens were treated with the toothpaste free of S-PRG fillers, few solid deposits can be seen on the dentin surface ( Figure 3a ) and inside the dentinal tubules ( Figure 3c ). Yet, after the erosive challenge, these deposits were mostly removed from the surface ( Figure 3b ) and from the dentinal tubules ( Figure 3d ). The same occurred for the specimens treated with toothpastes containing 1 wt.% ( Figure 4 ) and 5 wt.% of S-PRG fillers, with a few more deposits in the latter ( Figure 5 ).

For the specimens treated with toothpastes containing 20 wt.% ( Figure 6 ) and 30 wt.% ( Figure 7 ) of S-PRG fillers, the solid deposits on the dentin surface was higher than the previous groups. After the erosive challenge, however, these deposits were almost completely removed from the dentin surface ( Figures 6b and 7b ) and from the aperture of the dentinal tubules ( Figures 6d and 7d ).

Moreover, the amount of deposits on the surface was slightly greater for the specimens treated with the 1,450 ppm NaF toothpaste ( Figure 8a ). Yet, the amount of deposits decreased after an erosive challenge ( Figure 8b ).

After being treated with the 22,600 ppm NaF varnish ( Figure 9 ), the specimens presented a much higher amount of solid deposits on the dentin surface ( Figure 9a ) and at the aperture of the dentinal tubules ( Figure 9c ). The amount of deposits decreased after the erosive challenge both on the surface ( Figure 9b ) and at the apertures ( Figure 9d ), but it was still higher than the previous groups.

## Discussion

The results of this *in vitro* study indicate that the NaF varnish was able to reduce dentin permeability and differed from all S-PRG-based toothpastes. The NaF toothpaste and the one containing 5 wt.% were able to reduce the Lp after treatment, but their LpTreat values did not differ from the 20 wt.% and 30 wt.% of S-PRG fillers. Other concentrations of S-PRG fillers were not able to influence on the dentin permeability *in vitro* . Therefore, the null hypothesis was rejected.

Notably, our study focused on the S-PRG concentration itself, thus, the same composition was used for all tested toothpaste to avoid biases on the interpretation of the results. Therefore, this study contributes to the scientific literature by evidencing that the S-PRG technology available in different dental materials may contribute to controlling the symptoms of dentin hypersensitivity. Other published studies have been conducted addressing dentin permeability after the application of several fluoridated and non-fluoridated agents; ^15 , 17 - 19 , 23 , 24 , 28 , 30 - 33^ however, to this date, none of them have tested the efficacy of S-PRG-containing toothpastes with this purpose.

Based on the hypothesis that the acute pain sensation is due to the movement of fluid inside the tubules, leading to the stimulation of nerve cells, the design of this study considered different conditions of dentin. The LpMin represents a dentin covered with smear layer, in which the Lp value is below 25% of its maximum permeability. Then, after acid etching, the smear layer is removed, increasing the permeability by unclogging the aperture of dentinal tubules. Calabria, et al. ^24^ (2014) reported that a desensitizing effect is expected when the Lp after treatment (LpTreat) is similar to the LpMin values. However, in our study, the LpTreat differed from LpMin in all groups, regardless of the presence of S-PRG fillers.

In the study performed by João-Souza, et al. ^18^ (2019), no reduction of dentin permeability was seen after the application of any of the tested desensitizing toothpastes. These toothpastes may induce the deposition of some particles on the tooth surface and reduce the lumen of the tubules, which consequently reduce the flow of fluids inside the tubules to the power of 4, according to the Poiseuille’s law. ^18^ these deposits, however, may not be strongly bonded to the tooth structure and can be removed from the surface by rinsing or by the pressure of the water imposed by the Flodec device. ^18^

Other studies have also evidenced that tooth brushing promotes the formation of smear layer on the tooth structure, which is also able to interfere on the obliteration of dentinal tubules depending on the abrasiveness of toothpastes and the hardness of toothbrush bristles. ^34^ Since our study aimed to evidence the effect of the toothpastes on the dentin samples and not the toothbrush effect, toothpastes were applied using a disposable microbrush. Moreover, future investigations addressing the abrasive potential of S-PRG-based toothpastes should be conducted.

One dental alteration that may lead to dentin hypersensitivity is erosive tooth wear, in which layers of sound structure are dissolved. Even after treatment, patients are still commonly subjected to periodical erosive episodes, which result in the dissolution of the clogging particles and consequent opening of the tubules, directly increasing dentin hypersensitivity. ^24 , 30^ Additionally, acidic challenges are able to dissolve some fluoride deposits on top of tooth structure, shortening the desensitizing effect of fluoridated products, and some toothpastes are also known to increase the wear of eroded dentin. ^35^ Therefore, based on the fact that LpEro was statistically similar to LpMax in all groups in which a toothpaste was used, it can be suggested that the tested toothpastes were not able to prevent further unclogging of the dentinal tubules, hence, their desensitizing effect might not last an erosive challenge.

The only material that presented LpEro lower than LpMax was the NaF varnish. This might have occurred because the product was applied for a longer period (4 min), has a lower pH, and has a fluoride concentration more than fifteen times higher than that of the NaF toothpaste. Similarly to a previous study, ^24^ the NaF varnish was applied onto the specimen for a longer period since it is an in-office product, contrary to the toothpastes, which are at-home products and were applied for a shorter period (1 min and 15 s). However, despite LpEro being significantly different from LpMax, the LSCM analysis indicate that the amount of deposits formed on the specimens treated with the NaF varnish were significantly diminished after the erosive challenge. This might have occurred because the NaF varnish induces the formation of CaF _2_ deposits, which are not as acid resistant as fluoride varnishes containing polyvalent metals, such as TiF _4_ . ^25 , 36^ Moreover, despite the longer period (4 min) used to apply the NaF varnish, this product’s manufacturer recommend applying this product for 4 hours, which leads to a greater deposition of fluoride onto the tooth surface and further reduction of the permeability of dentin, suppressing the symptoms of dentin hypersensitivity more efficiently, especially considering the clinical effects that human saliva has.

As reported by Kaga, et al. ^9^ (2014), the S-PRG fillers are able to release ions upon pH decrease. ^6 , 9^ Among these ions, strontium and fluoride are able to reinforce the tooth structure by creating strontium-apatite and fluorapatite, which are more acid-resistant than conventional apatite. ^6 , 9^ Nevertheless, the formation of calcium fluoride-like precipitates related to tubules occlusion is dependent on fluoride concentration, pH, and time. ^37 , 38^ Thus, given that the pH of these toothpastes are within the neutral range and that the amount of fluoride released might be low, especially in the toothpastes with less than 5 wt.% of S-PRG fillers, this could justify the lack of reduction in dentin Lp. Also, this study applied the toothpaste only once, and subsequent applications of the product could also be responsible to improve its clinical efficacy.

Other important ion present in the S-PRG technology is Al ^3^ . This ion promotes tubule occlusion and reduces dentin permeability *in vitro* . ^26^ Yet, this potential requires further testing to be confirmed.

Nonetheless, considering that the specimens were rinsed after the application of the toothpaste and that the Flodec device is based on the passage of water through the dentin sample, the S-PRG fillers could have been washed away before the acidic condition (LpEro) was achieved. This is confirmed by figures obtained by LSCM in the groups in which a S-PRG-based toothpaste was applied. To prevent the fillers from being washed from the surface, other bioactive glasses contain polymers on their surface to bind to the dentin surface, increasing their retention and efficacy. ^39^

In the study of Spinola, et al. ^12^ (2020), the S-PRG fillers were inserted in different concentrations in a varnish to prevent enamel demineralization. The results have shown that the highly concentrated (30 wt.% and 40 wt.%) S-PRG-based varnishes were able to prevent enamel demineralization similarly to a 5% NaF varnish. The difference between that study and our study may be due to the fact that the varnish contains binding agents that help retain the varnish and the S-PRG fillers on the tooth surface for longer periods, resulting in an improved performance. ^12^ Moreover, considering that our study evaluated their effect on dentin, its higher complexity might also influence on the performance of these products compared to the enamel.

As observed in the study of Amaechi, et al. ^11^ (2018), toothpastes containing S-PRG were more effective in preventing tooth demineralization and in improving remineralization in the enamel than a conventional toothpaste containing 1,100 ppm of NaF. Moreover, in their study, the 5 wt.% concentration seemed to be the optimal concentration for toothpastes, given that it was not different from the most concentrated ones (20 wt.% and 30 wt.%). Despite having different objectives and evaluating different outcomes, their results differ from the ones in our study, in which the NaF toothpaste behaved similarly to the 5 wt.%, 20 wt.%, and 30 wt.% S-PRG-based toothpastes.

Furthermore, dentin samples are expected to have different levels of permeability depending on the depth of dentin, on the patient’s age, presence of previous tooth erosion or dental caries, as well as other genetic conditions, such as molar incisor hypomineralization, which is also related to the presence of dentin hypersensitivity. ^31^ Therefore, this study exclusively standardized the dentin depth of sound caries-free molars before the conductance of the study, with no signs of the abovementioned alterations. Nevertheless, our study was conducted on medium-depth dentin, which might exacerbate the clinical scenario regarding the mechanism of dentin hypersensitivity, given that patients often feel pain even under subclinical expositions of dentin to the oral environment. Thus, this *in vitro* model may not faithfully represent what occurs clinically, but it is relevant to discuss the mechanism of action of these products. This can be corroborated by the fact that the NaF varnish has already shown good results in suppressing the symptoms of dentin hypersensitivity clinically, similarly to other at-home and in-office products. ^17^ Since this methodology is used to measure and compare permeability against obliteration, it seems relevant to point out this limitation before translating these results to clinical circumstances.

Further studies should include some of the factors disregarded in this study, for instance human saliva, which plays a role in neutralizing acidic conditions and can also have some effect on tubule occlusion due to the formation of the acquired pellicle ^18^ and also by inducing the precipitation of minerals within the dentinal tubules. ^40^ Human saliva was not used in our study so as to analyze only the effect of the S-PRG fillers in the presence of deionized water. However, since human saliva works as a source of calcium and phosphate, the presence of these ions could have led to an increased formation of CaF _2_ deposits on the dentin surface, and consequent reduction of the specimens’ Lp values. ^28^ As dentin hypersensitivity impairs the quality of life of patients, technologies as S-PRG could enhance the potential and longevity of the products and should be further tested using other experimental models, including clinical studies.

## Conclusion

Within the limitation of this study, we can be conclude that a better reduction of the Lp was noticed for the NaF varnish. The toothpastes containing 5wt.%, 20wt.%, and 30wt.% of S-PRG fillers behaved similarly to a conventional NaF toothpaste. Despite also being affected by the erosive challenge, the NaF varnish was the only group that did not reach its LpMax values.
